# Imaging Multidimensional Therapeutically Relevant Circadian Relationships

**DOI:** 10.1155/2009/231539

**Published:** 2009-08-16

**Authors:** Jamil Singletary, Patricia A. Wood, Jovelyn Du-Quiton, Song Wang, Xiaoming Yang, Shobhit Vishnoi, William J. M. Hrushesky

**Affiliations:** ^1^Medical Chronobiology Laboratory, WJB Dorn VA Medical Center, University of South Carolina, SC 29209, USA; ^2^School of Medicine, University of South Carolina, SC 29209, USA; ^3^School of Public Health, University of South Carolina, SC 29209, USA; ^4^College of Engineering and Computing, University of South Carolina, SC 29209, USA; ^5^Center for Colon Cancer Research, University of South Carolina, SC 29209, USA

## Abstract

Circadian clocks gate cellular proliferation and, thereby, therapeutically target availability within proliferative pathways. This temporal coordination occurs within both cancerous and noncancerous proliferating tissues. The timing within the circadian cycle of the administration of drugs targeting proliferative pathways necessarily impacts the amount of damage done to proliferating tissues and cancers. Concurrently measuring target levels and associated key pathway components in normal and malignant tissues around the circadian clock provides a path toward a fuller understanding of the temporal relationships among the physiologic processes governing the therapeutic index of antiproliferative anticancer therapies. The temporal ordering among these relationships, paramount to determining causation, is less well understood using two- or three-dimensional representations. We have created multidimensional multimedia depictions of the temporal unfolding of putatively causative and the resultant therapeutic effects of a drug that specifically targets these ordered processes at specific times of the day. The systems and methods used to create these depictions are provided, as well as three example supplementary movies.

## 1. Introduction

### 1.1. Daily Temporal Organization

 Circadian organization of living organisms is ubiquitous. Within these organisms, circadian rhythms in synthetic, metabolic, and proliferative functions characterize all organs, tissues, and cells. This temporal organization is endogenous and conferred at all levels of biological integration. The mammalian central circadian clock, residing in the suprachiasmatic nuclei (SCN), sets circadian clocks throughout the body through autonomic nervous connections, SCN-orchestrated endocrine secretions, and complex SCN-influenced neurobiological behavior patterns such as human nightly sleep and daily activity [1–3]. Each cell in the body resonates with its own circadian orientation, determined by the relationship between the central time keepers' and its own molecular circadian clockwork [4–6]. 

 The molecular timekeeping components and mechanisms of the central and peripheral circadian clocks are apparently identical and comprised of the coordinated expression of some nine core circadian clock genes [6]. These core clock genes function in two linked feedback loops—one positive, the second negative. Traverse of these loops requires approximately 24 hours, creating a cellular circadian pacemaker, in principle, akin to the sinoatrial node in the heart. The phase and output of each peripheral clock are unique to that specific tissue. The time structure of each tissue clock is offset characteristically for that tissue from the central SCN-based circadian time structure. There is evidence that each tissue coordinates most of its key tissue-specific functions during each day through peripheral circadian clock gating of a unique set of tissue-specific circadian clock-controlled genes. At the gene expression level, these circadian clock-controlled genes comprise five to ten percent of the genome within each tissue [7]. 

 The processes linked to the clock and the clock-controlled genes are unique for each tissue. For example, the liver organizes albumin synthesis, bile metabolism, and detoxification pathways within each day, while the bone marrow and gut cells most prominently order cell proliferation [8, 9]. When the liver is injured or resected, however, hepatocyte regenerative proliferation is promptly induced. This “on-demand” liver cell proliferation cassette is tightly gated during each day by the identical clock and clock-controlled genes that order proliferation in other constitutively dividing tissues [10, 11]. 

### 1.2. Cancers Tell Time

 Cancers in human beings and mice are characterized by abnormal balance among cancer cell proliferation, dormancy, and apoptosis. Since these physiologic states are clocked within each normal tissue, it is likely that the development of cancer requires some disordering of these clock-controlled states [12]. We have been surprised, therefore, to learn that cancer cell proliferation still remains tightly circadian-controlled in some human and mouse cancers [13, 14]. This temporal circadian organization of cancer cell proliferation provides a therapeutic opportunity for optimally timed cancer treatments to diminish side effects as well as to maximize anticancer activity in cancer patients [15]. 

### 1.3. Cancer Control Depends upon Treatment Timing

Since many anticancer agents target proliferative pathways and since cancer cell proliferation is circadian-rhythmic, anticancer drug target availability within living cancer cells is necessarily largely organized during each day. Randomized clinical trials have demonstrated that this circadian organization is evident even among genetically and environmentally distinct cancer patients and can be used to improve the outcome of cancer treatment. The judicious timing of antiproliferative anticancer agents during the day resulted in improved survival of women with metastatic ovarian cancer and increased metastatic colon cancer objective tumor response frequency [16, 17]. At the same time, optimal drug timing diminishes the toxicity of these agents to the host gut and bone marrow several fold [18, 19]. These therapeutic advantages accrue despite the fact that the patients studied in these randomized clinical trials and their cancers are genetically distinct from one another and despite the fact that patients with advanced cancer are ill.

## 2. Methods

A series of experiments was performed in 10- to 14-week old CD2F1 female mice, kept on a 12-hour light: 12-hour dark light regimen from birth. Time of day is referred to as Hours After Lights On (HALO). Tumors were established by transplantation, subcutaneously injected into the mouse flank with 5 × 10^5^ viable meth-A sarcoma cells (provided by the laboratory of Dr. Lloyd Old). When tumor nodules were palpable, tumors were measured daily. When these tumors averaged 500 mm^3^ in volume, mice were either: (a) killed at one of six equispaced consecutive times of day (every 4 hours) to harvest tumor and host tissues, or (b) treated with intravenous 5-flourouracil (5-FU) chemotherapy drug at an effective dose at one of these same six times of day. Daily tumor measurements were continued and mice were followed until death [20]. 

 These tumor-bearing mice killed around the clock had bone marrow, intestinal epithelium, and tumor tissues removed, bisected, and stored at −80°C. The other half of bisected tumors were fixed in 10% buffered formalin for 24 hours and embedded in paraffin for subsequent staining and microscopic examination and immunohistochemical analysis. Thymidylate synthase (TS), an enzyme which, in the presence of folate and substrate, produces thymidylate, without which DNA cannot be synthesized, is the primary target of 5-FU antiproliferative effect. TS message, protein, and enzymatic activity were each measured in intestinal mucosa, bone marrow, and tumor samples obtained every 4 hours around a 24-hour period. Mitotic index, growth factor VEGF protein content, clock protein BMAL-1 nuclear and cytoplasmic content, and WEE1 protein (which gates mitosis) content were also measured in every tumor sample. 

 The tumor-bearing mice treated with 5-FU had measurements of daily serial tumor size, tumor disappearance rates, and survival assessments to determine therapeutic effects. 5-FU toxicity to healthy host cells was assessed by body weight fall and recovery, white blood cell fall and recovery, and perianal swelling (GI toxicity). A toxic-therapeutic index (TI) was then calculated for each 5-FU-treated mouse by combining the resulting drug toxicity to the host and tumor therapeutic efficacy scores. High TI values indicated a simultaneous occurrence of low host 5-FU toxicity with high anti-tumor therapeutic effects. Low TI values indicated high host 5-FU toxicity and low anti-tumor therapeutic effects. 

 These measurements allowed us to determine the circadian relationships among tumor growth, relevant tumor cell clock function, 5-FU target availability in normal and cancer tissues, and the circadian organization of the resultant 5-FU therapeutic index (anticancer efficacy and bone marrow and gut toxicity). 

### 2.1. Multidimensional Processes

 Movies were created in the form of 3-dimensional (3D) contour plots of three potential causally relevant factors at a time using bivariate interpolated values through Proc G3grid (SAS version 12, Cary, NC). The procedure creates a data set where the horizontal (*x*) and vertical (*y*) variable values form a complete grid, and it interpolates the value of the depth (*z*) variable for each point on the *x*-*y* plane. Assuming that the relationship between two times of day (HALO) measurements is linear, data obtained from two consecutive times of day (e.g., 2 and 6 HALO) were then linearly interpolated to create data that span about every 8 minutes between these two times of actual measurement. 

 In order to interpolate these data points, the following formula was used:

(1) $$\frac{H_{2}-H_{1}}{n}+P_{1}.$$

In Formula (1), *H*_1_ refers to the value at a marked (measured) HALO point, *H*_2_ refers to the following data value at the next marked HALO point (4 hours later), *n* refers to the number of time points to be interpolated, and *P*_1_ refers to the data value from the previous time point (8 minutes prior). For these sets of data, 30 was chosen as the number of data points, interpolated between each actual physical and biochemical measurement.

 Upon completion of the linear interpolation method, the end result is a spreadsheet of values that span 121 rows and 181 columns of data values for each of these biologically relevant variables. We then use Co-Plot Software (Monterey, CA) to create 3D graphs, creating an 8-minute interval frame over a 24-hour span which is sequentially displayed to create movies depicting the 3D relationship over circadian time. 

 We have created three movies visualizing the relationships of an undulating surface changing over the 24-hour circadian cycle, comprised of the beating circadian clock as created from quantitative measurement of BMAL-1 clock protein in the tumor cell nucleus where it acts to keep time; the maximum amount of tumor shrinkage occurs as a result of circadian-timed 5-FU administration; the 5-FU Toxic-Therapeutic Index (TI) with (1) cell DNA replication, (2) cancer cell division and growth, and (3) 5-FU target, toxicity, and therapeutic effects. The undulating 3D surface relationships turn from blue (best), to purple (intermediate), to red (worst), to purple, to blue as TI changes rhythmically over circadian time. Because the rates of change of these relationships are also rhythmically changing throughout each day, a musical score is added in two acoustic dimensions. The melody cycles once throughout each completed circadian cycle. The beat/cadence of the musical score quickens as the changes in the relationships over these 24 hours accelerate and slows as they decelerate. 

 Finally, inspirational major chords accompany favorable circadian stages while melancholy minor chords accompany biomedically unfavorable relationships. Harmonically-favorable circadian stages are independently calculated by quantifying the survival duration of tumor-bearing mice treated at six circadian stages with 5-FU. This shape, color, and audio scheme concurrently depict the relationships among tumor cell circadian clock function, circadian tumor cell availability of the target of 5-FU, TS activity (TSA), the circadian therapeutic pattern of 5-FU induced tumor shrinkage, the TI of 5-FU treatment and the rate of change of these relationships within the day, and the overall survival benefit for tumor-bearing mice resulting from 5-FU treatment at six times of day.

## 3. Results

### 3.1. Relevant Previous Findings

We have previously described the chain of events linking cancer cell clock proteins, cancer cell DNA synthesis, proliferation, TSA, and 5-FU TI to explain the dependence of cancer outcome on circadian timing of 5-FU [20]. We have shown that despite the fact that cancers often exhibit deregulated cellular proliferation, cancers maintain high amplitude circadian rhythms in their growth, DNA synthesis, and mitosis. Circadian-timed tissue acquisition allowed us to determine circadian relationships among tumor size, relevant clock and clock-controlled proteins, and 5-FU drug target availability (e.g., TS) in normal and cancer tissues. A separate study of circadian-timed 5-FU drug administration allowed us to determine circadian relationships among drug-induced host bone marrow and gut toxicity, tumor shrinkage, and the balance between 5-FU toxicity and anti-tumor effects (e.g., TI). The time of day was recorded as Hours After Lights On (HALO). The time of day (HALO) at which tumor TSA is low, 5-FU TI is high (See supplementary Tables 1 and 2 in Supplementary Material avialable at doi:1155/2009/231539). At this time of day, tumor WEE-1 and nuclear BMAL1 levels are higher (Supplementary Table 3). Conversely, HALOs at which tumor TSA is high, 5-FU TI is low. At these times, tumor WEE-1 and nuclear BMAL1 are lower [20].

### 3.2. 3-D Movies Uncover Diurnal Relationships

Understanding the temporal relationships of the circadian dynamics of cancer and normal cell clock function, DNA synthetic activity, mitosis, tumor growth rate, and cancer drug therapeutic index is challenging. We have therefore created a series of multidimensional motion pictures to describe these predictable rhythmic relationships; each endpoint depicted is statistically significantly modulated during circadian time [20]. These movies show the relationship among molecules key to DNA synthesis, cell division and susceptibility of tumor and normal tissue to the effects of 5-FU. Each movie depicts, in two dimensions, the circadian differences in tumor volume or the toxic therapeutic index of 5-FU and tumor nuclear BMAL-1 clock protein content. Also added is a measurement of time kept by the external environment through the rising and setting of the sun and the clock in the left-hand corner.

#### 3.2.1. Cancer Cell DNA Replication

**Figure 1:**
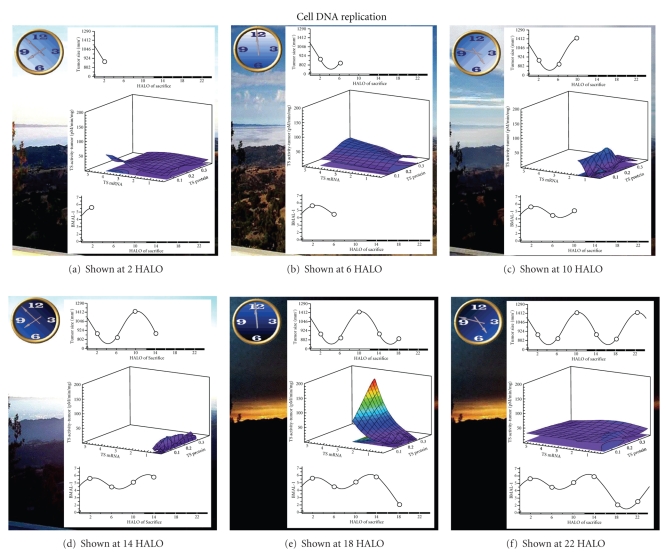
Snapshot of Supplementary Movie 1. The cytotoxicity of 5-FU (5 Fluorouracil), an agent targeting thymidylate synthase (TS), is greatest when TS activity is the lowest. Both message and protein are necessary precursors for TS enzyme activity. Average tumor size is the lowest between 2 and 6 Hours After Lights On (HALO) when TS message and protein are both present. TS activity rises modestly as tumor size increases. TS message then fades as TS protein persists, TS activity declines and tumor size decreases. TS activity then spikes followed by tumor size and TS activity recession with residual TS message and protein. Times of day associated with the lowest tumor cell enzyme activity are the times of day associated with greatest 5-FU tumor cell anticancer activity. Times of day associated with highest tumor cell TS activity correspond to times of day of greatest tumor cell resistance to the cytotoxic effects of 5-FU. Tumors can thereby be expected to be most resistant to 5-FU in the middle of the daily dark/activity span (18 HALO) and most sensitive to 5-FU in the middle of the daily light/sleep span.

DNA synthesis occurs during the S phase of the cell cycle and is accompanied by an increase in TSA. TS is the enzyme inhibited by 5-FU. Supplementary Movie 1 (see also Figure 1) shows the relationship of tumor size and tumor TS message, protein, enzyme activity, and tumor nuclear BMAL1 within the circadian cycle. This movie depicts that a high concentration of TS mRNA precedes high TS protein and TSA, which is expected as protein is preceded by mRNA. At 2 HALO, tumor mRNA concentration is at its peak which consequently leads to the maximum tumor TS protein at the next HALO (6 HALO), which is associated with the highest amount of TSA at 6 HALO. Then at 6 and 10 HALO, tumor TS mRNA levels decrease leading to lower tumor TSA levels at 10 and 14 HALO. Tumor TSA peaks at 6 and 22 HALO, which coincides with or just precedes the peak in tumor size, linking tumor growth and DNA synthesis throughout the circadian cycle. Nuclear BMAL1 daily rhythm is shown in the bottom panel as a measure of the circadian clockwork in tumor cells. The best 5-FU therapeutic outcome is achieved when tumor TSA levels are low (also see Figure 3). At 2 and 14 HALO, tumor size is low, which is also when nuclear BMAL1 is high. 

#### 3.2.2. Cancer Cell Division and Growth

**Figure 2:**
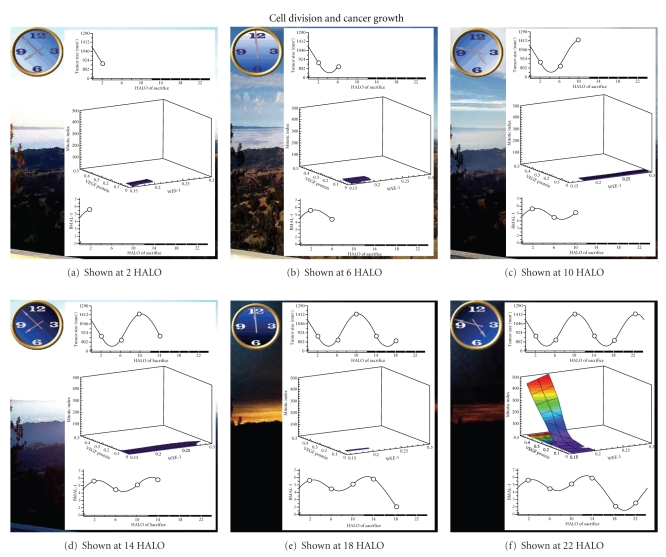
Snapshot of Supplementary Movie 2. WEE-1 is known to prevent or gate mitosis at the G_2_M interface of the cell cycle. VEGF is known to promote cancer growth and cancer cell division. Throughout the day as WEE-1 increases, no mitosis occurs. As WEE-1 decreases, mitosis appears and gradually increases to a high level. Concurrently, VEGF content of the same tumor cell increases markedly.

**Figure 3:**
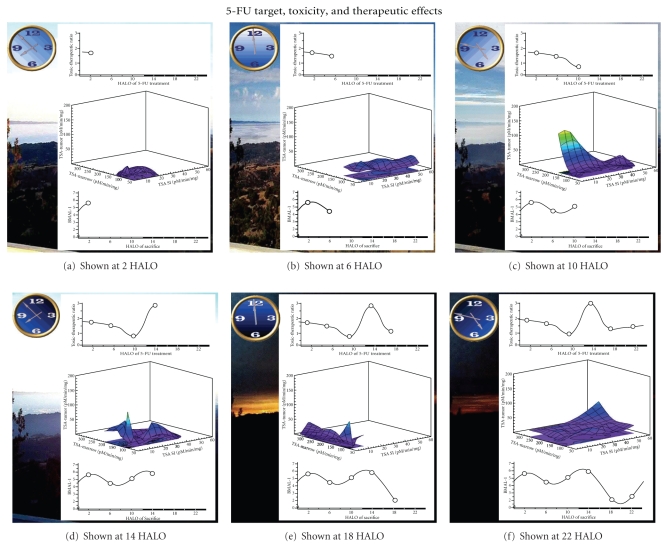
Snapshot of Supplementary Movie 3. The relationship between 5-FU Target and Toxic-Therapeutic ratio proceeds throughout the day indicating time of day association with low tumor thymidylate synthase activity are concurrent with times of day of the greatest anticancer activity. Times of low thymidylate synthase in bone marrow and gut cell are those same times of day associated with the greatest gut damage and bone marrow damage. Toxic-therapeutic ratio is maximum at times of day of relatively low tumor thymidylate synthase activity and relatively high bone marrow and gut TSA.

WEE-1 is a clock-controlled protein and inhibits and gates mitosis at the G_2_M interface of the cell cycle. Supplementary Movie 2 (see also Figure 2) shows the relationship of tumor size to tumor mitotic index (vertical axis), tumor cell VEGF (horizontal axis) and tumor WEE-1 (oblique axis) over the circadian cycle. Throughout the circadian cycle, tumor WEE-1 and tumor mitotic index (MI) have an inverse relationship. VEGF is a clock-controlled growth factor that promotes cancer growth. During the circadian cycle, tumor VEGF levels vary in parallel to tumor MI. This movie shows that as tumor WEE-1 increases, reaching its maximum in early activity phase (14 HALO), VEGF begins to increase and then drops, which is accompanied by a decrease in tumor MI. A decrease in WEE-1 during the late activity/ early sleep span is accompanied by a sudden surge in VEGF, at which time tumor MI levels increase. Poor 5-FU therapeutic outcomes are found at high tumor MI levels at 10 and 22 HALO, at which time tumor nuclear BMAL1 is lower (Figure 3). 

#### 3.2.3. 5-FU Effects on Both Tumor and Normal Cells

Supplementary Movie 3 (see also Figure 3) depicts the relationship of 5-FU toxic-therapeutic index (TI) to the amount of drug target (e.g., TSA) in the gut epithelium cells, nucleated bone marrow cells, and tumor cells. The most favorable outcome (high TI) is when tumor TSA is at its minimum, allowing tumor TS to be more easily inhibited by 5-FU. 5-FU will also be harmful to the host when TSA levels of healthy gut and bone marrow cells are low. Thus, the best therapeutic effect (harmful to the tumor cells but little effect on healthy cells) of 5-FU occurs when TSA in tumor cells is relatively low, at a time when TSA in gut epithelium and bone marrow cells are relatively high. TI is calculated by considering both the toxicity of 5-FU to the host (absolute AUC WBC counts, absolute AUC body weight, and presence or absence of perianal swelling) and anti-tumor therapeutic effects (tumor remission, decrease in tumor size, and survival). A higher value of TI demonstrates low host drug toxicity along with high tumor therapeutic effects. This movie depicts that the best outcome (highest TI level) is at 14 HALO and at 2 HALO. At 14 HALO, tumor TSA is at a minimum and, although small intestine TSA and bone marrow TSA are not at their maximum levels which occur at 6 HALO and 18 HALO respectively, they are still relatively high, shielding them from the effects of 5-FU. Thus, at 14 HALO, 5-FU creates the largest difference between toxicity to the tumor cells versus toxicity to gut and bone marrow cells. Figure 3 demonstrates that at 22 HALO, tumor TSA is high, which leads to a low TI. As shown, a high tumor TSA level is also associated with low nuclear BMAL1 levels. Depicted in blue in the middle graph is where TI is at a maximum, which occurs at 14 HALO with high levels of gut cell and bone cell TSA and concurrent low tumor TSA. 

## 4. Discussion

Being able to visualize and hear the diurnal/nocturnal dynamics of the host tissues and the cancer demonstrates how and why the circadian timing of 5-FU determines both its efficacy and its toxicity. These relationships are clinically relevant and have already been proven in metastatic colorectal cancer patients [21, 22]. 

 Obviously, our chosen methods have limitations. Linear interpolations between four-hourly observations make the unwarranted assumption that changes between the six measured circadian phases is unidirectional and continuous. These assumptions must be kept in mind while viewing the movies provided. 

 The data presented here contains a plethora of information that must be understood concurrently. Such a method can be useful in displaying other data that also change and evolve over time, linearly, rhythmically, or chaotically. By introducing other variables such as color scheme, music, and internal and environmental time measurements, we are able to present other dynamical cues. 

 Creating such a multimedia display aims to arouse each of the senses of the viewer to enhance understanding. We, herein, have developed a unique way to take a large number of ordered raw data and convert these into meaningful visual and aural experiences. In doing this, many obstacles have been encountered. Multiple pieces of software had to be used in order to optimally create these movies and integrating them was a challenge. 

 The major drawbacks of using multiple pieces of software are that our data must be converted to different file types throughout the entire process. At each such step, this creates potential for error. Therefore, we have begun to create a purpose built software package called the Temporal Data Transformer. The goal of the Temporal Data Transformer will be an easier, more effective “automatic” creation of such movies directly from the time coded temporal data stream.

**Commentary**

The most important test of causality is temporality. In order for an event to cause or contribute to the cause of another event, the causative event must precede the caused event. All biological processes are precisely ordered in time. This temporal organization is commanded by evolution for thermodynamic reasons, translated practically as essential biological economy and parsimony.

Time is measured solely by triangulation between and among recurrent events. Events and milieu favorable to the occurrence of complex biological events require opportunities for their recurrence as a prerequisite for life. Without modulation of time's merciless arrow by biological systems, life would be an impossibility. If biological systems truly could not step into the same river twice, life, at least as we know it, could not exist.

These opportunities for recurrence have been guaranteed by evolution in response to the time structures of our near cosmos. Cosmic cycles of profound regularity have been borrowed by all living things and used to organize all life processes rhythmically. The circadian frequency range is the most thoroughly explored. We and others have shown that cellular proliferation is one such complex life process that is tightly organized at the cellular and tissue levels within each day. We have shown this to be true in cancer cells and tumors.

This profound rhythmic circadian organization of all cellular events essential for proliferation makes the search for temporality complex but impossible if this time structure is ignored. If the cyclical nature of this essential aspect of life is ignored in favor of a linear approach, causality relationships will appear where they do not exist and be unapparent where they clearly exist. This is especially dangerous where experiments supposedly determining cause-and-effect are done outside of a resonating living organism.

We have begun to address this indirect complexity by making measurements of proliferation in key host and cancer tissues, around the circadian clock, and then displaying the resultant relationships visually and aurally, looped over the entire circadian span. We believe that this approach, which can be duplicated for any rhythmic time-coded data, may prove useful to those who wish to understand complex interactive cause-and-effect relationships that characterize all life including therapeutically relevant host-cancer relationships—the complex symphony of life without melody, tempo, and rhythm in apparent cacophony.

## Supplementary Material

Supplementary Table 1 shows the circadian variation in tumor size and thymidylate synthase in tumor and normal tissues.
Supplementary Table 2 shows the circadian-dependent 5-FU effects on host toxicity, anti-tumor efficacy and toxic-therapeutic index.
Supplementary Table 3 shows the circadian variation in tumor histopathology.
Supplementary Movie 1 depicts the relationship of tumor size and tumor TS message, protein, enzyme activity, and tumor nuclear BMAL1 within the circadian cycle.
Supplementary Movie 2 shows the relationship of tumor size to tumor mitotic index (vertical axis), tumor cell VEGF (horizontal axis) and tumor WEE-1 (oblique axis) over the circadian cycle.
Supplementary Movie 3 depicts the relationship of 5-FU toxic-therapeutic index (TI) to the amount of drug target (e.g. TSA) in the gut epithelium cells, nucleated bone marrow cells, and tumor cells.

## References

[1] Meijer JH, Rietveld WJ (1989). Neurophysiology of the suprachiasmatic circadian pacemaker in rodents. *Physiological Reviews*.

[2] Moore RY, Eichler VB (1972). Loss of a circadian adrenal corticosterone rhythm following suprachiasmatic lesions in the rat. *Brain Research*.

[3] Schwartz WJ, Gainer H (1977). Suprachiasmatic nucleus: use of 14C-labeled deoxyglucose uptake as a functional marker. *Science*.

[4] Moore RY, Lenn NJ (1972). A retinohypothalamic projection in the rat. *Journal of Comparative Neurology*.

[5] Obrietan K, Impey S, Storm DR (1998). Light and circadian rhythmicity regulate MAP kinase activation in the suprachiasmatic nuclei. *Nature Neuroscience*.

[6] Reppert SM, Weaver DR (2002). Coordination of circadian timing in mammals. *Nature*.

[7] Panda S, Antoch MP, Miller BH (2002). Coordinated transcription of key pathways in the mouse by the circadian clock. *Cell*.

[8] Buchi KN, Moore JG, Hrushesky WJM, Sothern RB, Rubin NH (1991). Circadian rhythm of cellular proliferation in the human rectal mucosa. *Gastroenterology*.

[9] Wood PA, Hrushesky WJM, Klevecz R (1998). Distinct circadian time structures characterize myeloid and erythroid progenitor and multipotential cell clonogenicity as well as marrow precursor proliferation dynamics. *Experimental Hematology*.

[10] Barbason H, Smoliar V, Van Cantfort J (1979). Correlation of liver growth and function during liver regeneration and hepatocarcinogenesis. *Archives of Toxicology*.

[11] Matsuo T, Yamaguchi S, Mitsui S, Emi A, Shimoda F, Okamura H (2003). Control mechanism of the circadian clock for timing of cell division in vivo. *Science*.

[12] Hrushesky WJM (2000). The temporal organization of life: the impact of multi-frequency non-linear biologic time structure upon the host-cancer balance. *Japanese Journal of Clinical Oncology*.

[13] You S, Wood PA, Xiong Y, Kobayashi M, Du-Quiton J, Hrushesky WJM (2005). Daily coordination of cancer growth and circadian clock gene expression. *Breast Cancer Research and Treatment*.

[14] Hrushesky WJM, Lannin D, Haus E (1998). Evidence for an ontogenetic basis for circadian coordination of cancer cell proliferation. *Journal of the National Cancer Institute*.

[15] Hrushesky WJM, Fader D, Schmitt O, Gilbertsen V (1984). The respiratory sinus arrhythmia: a measure of cardiac age. *Science*.

[16] Lévi F, Zidani R, Misset J-L (1997). Randomised multicentre trial of chronotherapy with oxaliplatin, fluorouracil, and folinic acid in metastatic colorectal cancer. *The Lancet*.

[17] Hrushesky WJM, Bjarnason GA (1993). Circadian cancer therapy. *Journal of Clinical Oncology*.

[18] Gruber S, Cipolle R, Canafax D, Reinberg A, Smolensky M, Labrecque G (1988). Circadian-shaped intrarenal cyclosporine delivery. *Annual Review of Chronopharmacology*.

[19] Hrushesky WJM (1985). Circadian timing of cancer chemotherapy. *Science*.

[20] Wood PA, Du-Quiton J, You S, Hrushesky WJM (2006). Circadian clock coordinates cancer cell cycle progression, thymidylate synthase, and 5-fluorouracil therapeutic index. *Molecular Cancer Therapeutics*.

[21] Lévi F, Giacchetti S, Adam R, Zidani R, Metzger G, Misset J-L (1995). Chronomodulation of chemotherapy against metastatic colorectal cancer. International Organization for Cancer Chronotherapy. *European Journal of Cancer A*.

[22] Lévi F, Zidani R, Misset J-L (1997). Randomised multicentre trial of chronotherapy with oxaliplatin, fluorouracil, and folinic acid in metastatic colorectal cancer. International Organization for Cancer Chronotherapy. *The Lancet*.

