# Supplementary material for: Imaging Multidimensional Therapeutically Relevant Circadian Relationships
Source: Int J Biomed Imaging. 2009 Aug 16;2009:231539. doi: 10.1155/2009/231539 (PMC2727657; doi:10.1155/2009/231539)
Supplement: Supplementary file 1 — Supplementary Table 1 shows the circadian variation in tumor size and thymidylate synthase in tumor and normal tissues. Supplementary Table 2 shows the circadian-dependent 5-FU effects on host toxicity, anti-tumor efficacy and toxic-therapeutic index. Supplementary Table 3 shows the circadian variation in tumor histopathology. Supplementary Movie 1 depicts the relationship of tumor size and tumor TS message, protein, enzyme activity, and tumor nuclear BMAL1 within the circadian cycle. Supplementary Movie 2 shows the relationship of tumor size to tumor mitotic index (vertical axis), tumor cell VEGF (horizontal axis) and tumor WEE-1 (oblique axis) over the circadian cycle. Supplementary Movie 3 depicts the relationship of 5-FU toxic-therapeutic index (TI) to the amount of drug target (e.g. TSA) in the gut epithelium cells, nucleated bone marrow cells, and tumor cells. [file 231539.f1.doc]

**Supplementary Table 1. Circadian Variation in Tumor Size and Thymidylate Synthase in Tumor and Normal Tissues.**

**2**

**6**

**10**

**14**

**18**

**22**

**Fc**

**p-value**

**Rhythm**

**p-value**

**Circadian Peaks**

**Fitted(hr)**

**(HALO)**

**Tumor size (mm^3^)**

**988±96**

**753±71**

**1073±82**

**783±87**

**901±67**

**1292±119**

**5.22**

**<0.001**

**12**

**<0.001**

**10:30 ; 22:30**

**24**

**0.080**

-

**12&24**

**<0.001**

**10:30 ; 22:30**

**Thymidylate Synthase Activity**

**Tumor**

**a**

**17.8±1.8**

**26.9±1.8**

**17.7±2.1**

**15.1±1.9**

**16.7±1.7**

**23.7±2.0**

**6.26**

**<0.001**

**12**

**0.018**

**7:30 ; 19:30**

**24**

**0.003**

**3:00**

**12&24**

**<0.001**

**7:00 ; 20:00**

**Small Intestine**

**a**

**6.7±1.1**

**27.9±3.0**

**25.2±2.9**

**17.0±4.2**

**6.5±0.6**

**17.9±2.7**

**11.58**

**<0.001**

**12**

**0.012**

**9:00 ; 21:00**

**24**

**<0.001**

**8:30**

**12&24**

**<0.001**

**9:00 ; 21:00**

**Bone Marrow**

**a**

**85.8±8.6**

**106.8±7.5**

**127.2±6.7**

**163.6±12**

**184.5±16.3**

**148.7±8.6**

**11.25**

**<0.001**

**12**

**0.260**

**-**

**24**

**<0.001**

**17:00**

**12&24**

**<0.001**

**7:00 ; 18:00**

**Tumor Thymidylate Synthase (TS)**

**TS Protein**

**b**

**1.26±0.32**

**1.37±0.35**

**0.56±0.10**

**0.27±0.04**

**0.74±0.20**

**1.73±0.43**

**3.36**

**0.008**

**12**

**0.713**

**-**

**24**

**<0.001**

**1:00**

**12&24**

**0.002**

**7:30 ; 23:30**

**TS mRNA**

**c**

**0.24±0.03**

**0.16±0.03**

**0.17±0.03**

**0.12±0.12**

**0.17±0.02**

**0.22±0.04**

**1.79**

**0.133**

**12**

**0.62**

**-**

**24**

**0.034**

**0:30**

**12&24**

**0.102**

**-**

^a^pM/min/mg; ^b^Optical density (sample/standard); ^c^TS/18S (%phosphorimage units). Table reproduced from Wood, P.A., Du-Quiton, J., You, S. and Hrushesky, W.J.M. (2006) Circadian clock coordinates cancer cell cycle progression, thymidylate synthase, and 5-fluorouracil therapeutic index, *Mol Cancer Ther*, **5**, 2023-2033. Copyright 2006, Molecular Cancer Therapeutics.

**Cosinor Analysis**

**ANOVA**

**Time of day (HALO) of Sacrifice**

**mean±se**

**Supplementary Table 2. Circadian Dependent 5-FU Effects on Host Toxicity, Anti-Tumor Efficacy and Toxic-Therapeutic Index.**

**2**

**6**

******

**10**

**+**

**14**

*****

**18**

**22**

**++**

**Test stat**

**a**

**p-value**

**Rhythm**

**p-value**

**Circadian Peak**

**Fitted(hr)**

**(HALO)**

**Anti-Tumor Efficacy**

**Tumor Response**

**51.7±8.0**

**34.9±7.2**

**71.9±14.8**

**31.2±5.7**

**48.1±5.2**

**65.1±14.6**

**2.59**

**0.032**

**12**

**0.010**

**10:00 ; 22:00**

**(Treated/Control)**

**b**

**24**

**0.765**

**-**

**12&24**

**0.048**

**10:00 ; 22:00**

**Tumor Remission**

**1 CR**

**2CR, 1C**

**Survival**

**Time to death (days)**

**24.9±1.8**

**27.6±2.4**

**21.3±2.3**

**36.5±3.3**

**27.6±2.4**

**26.8±2.9**

**3.96**

**0.003**

**12**

**0.048**

**3:00 ; 15:00**

**24**

**0.103**

**-**

**12&24**

**0.028**

**3:00 ; 15:30**

**Host Toxicity**

**WBC AUC**

**18.3±1.5**

**24.6±4.3**

**47.1±6.1**

**26.1±3.4**

**18.8±1.9**

**22±1.8**

**8.88**

**<0.001**

**12**

**0.004**

**10:00 ; 22:00**

**(Absolute, Day 0-6, x1000)**

**24**

**<0.001**

**10:00**

**12&24**

**<0.001**

**10:00 ; 22:00**

**Body Weight AUC**

**12±1.5**

**12.4±1.1**

**16.5±1.6**

**10.0±1.0**

**12.4±1.8**

**15.8±1.6**

**2.95**

**0.017**

**12**

**0.002**

**9:30 ; 21:30**

**(Absolute, Day 0-6)**

**24**

**0.749**

**-**

**12&24**

**0.010**

**9:30 ; 21:30**

**Peri-anal Swelling**

**4/15(27)**

**7/15(47)**

**5/10(50)**

**3/15(20)**

**10/14(71)**

**2/15(13)**

**14.61**

**c**

**0.012**

**-**

**-**

**-**

**(affected/total animals, %)**

**Toxic-Therapeutic Index**

**d**

**103.6±4.5**

**108.3±4.4**

**72.9±5.4**

**117.2±2.3**

**103.5±4.1**

**93.5±6.2**

**7.1**

**<0.001**

**12**

**<0.001**

**4:30 ; 16:30**

**24**

**0.392**

**-**

**12&24**

**<0.001**

**4:30 ; 16:30**

^a^Analysis of Variance unless noted otherwise; ^b^6th day after 5-FU injection when first death is observed [(Tumor at Day6-Day0)/(Control at Day 6-Day0)]*100; ^c^based on a Chi-square test of independence; CR-complete response; C-cure; AUC- Area under the curve; *Most optimal time of day for both 5-FU t Toxic-Therapeutic Index is the sum of overall toxicity and tumor therapeutic scores calculated for individual mice (see Methods); ^d^Toxicity and anti-tumor efficacy was 14 HALO; ** with second optimal time of day at 6 HALO; ^+^Worst time of day for both 5-FU toxicity and anti-tumor efficacy was 10 and 22 HALO. Table reproduced from Wood, P.A., Du-Quiton, J., You, S. and Hrushesky, W.J.M. (2006) Circadian clock coordinates cancer cell cycle progression, thymidylate synthase, and 5-fluorouracil therapeutic index, *Mol Cancer Ther*, **5**, 2023-2033. Copyright 2006, Molecular Cancer Therapeutics.

**mean±se**

**Time of day (HALO) of 5FU Treatment**

**Statistical Analysis**

**Cosinor Analysis**

**Supplementary Table 3. Circadian Variation in Tumor Histopathology.**

**2**

**6**

**10**

**14**

**18**

**22**

**Fc**

**p-value**

**Rhythm**

**p-value**

**Circadian Peaks**

**Fitted(hr)**

**(HALO)**

**VEGF protein**

**a**

**4.42±0.9**

**5.08±0.5**

**3.93±0.5**

**3.57±0.6**

**6.97±0.6**

**5.93±0.7**

**3.51**

**0.009**

**12**

**0.044**

**7:00 ; 19:00**

**24**

**0.025**

**21:30**

**12&24**

**0.004**

**7:00 ; 19:30**

**Mitotic Index**

**2.68±0.4**

**5.36±0.8**

**5.16±0.4**

**4.93±0.5**

**3.22±0.5**

**4.17±0.5**

**4.6**

**0.001**

**12**

**0.233**

**-**

**24**

**0.009**

**10:00**

**12&24**

**0.012**

**9:00 ; 21:00**

**BMAL 1 Nuclear Protein (x10)**

**a**

**5.6±0.7**

**4.6±0.5**

**5.1±0.8**

**6.0±0.5**

**2.1±0.4**

**2.8±0.3**

**7.19**

**<0.001**

**12**

**0.004**

**1:30 ; 13:30**

(positive nuclear/total nuclear area)

**24**

**0.003**

**8:30**

**12&24**

**<0.001**

**1:30 ; 12:30**

**BMAL 1 Total Protein (x10)**

**a**

**1.8±0.1**

**1.9±0.01**

**1.9±0.1**

**1.9±0.1**

**1.8±0.01**

**1.9±0.1**

**0.75**

**0.590**

**-**

**NS**

**-**

**Wee1 Total Protein (x100)**

**a**

**12.0±1.2**

**14.0±1.1**

**14.2±1.5**

**17.9±0.8**

**10.5±1.8**

**12.8±1.3**

**3.32**

**0.011**

**12**

**0.325**

**-**

**24**

**0.047**

**11:30**

**12&24**

**0.060**

**1:00; 12:30**

a

Optical Density per high power field; NS - not significant; HALO=Hours after Lights Out on 12hr/12hr light: dark lighting schedule. Table reproduced from Wood, P.A., Du-Quiton, J., You, S. and Hrushesky, W.J.M. (2006) Circadian clock coordinates cancer cell cycle progression, thymidylate synthase, and 5-fluorouracil therapeutic index, *Mol Cancer Ther*, **5**, 2023-2033. Copyright 2006, Molecular Cancer Therapeutics.

**Time of day (HALO) of Sacrifice**

**ANOVA**

**Cosinor Analysis**

**mean±se**
